# Exosomes in pathogenesis, diagnosis, and treatment of pulmonary fibrosis

**DOI:** 10.3389/fphar.2022.927653

**Published:** 2022-08-25

**Authors:** Yang Yang, Yufei Liu, Yilu Chai, Ke Liu, Wei Hu, Keni Zhao, Yi Zhu, Peiyang Gao, Qingsong Huang, Chuantao Zhang

**Affiliations:** ^1^ Department of Respiratory Medicine, Hospital of Chengdu University of Traditional Chinese Medicine, Chengdu, China; ^2^ Department of Critical Care Medicine, Hospital of Chengdu University of Traditional Chinese Medicine, Chengdu, China

**Keywords:** exosomes, pulmonary fibrosis, pathogenesis, therapeutics, biomarkers

## Abstract

Pulmonary fibrosis (PF) is a group of interstitial lung diseases that seriously endanger human life and health. Despite the current advances in research on the pathogenesis and treatment of PF, the overall quality of survival and survival rates of PF patients remain low, prompting the search for more effective therapeutic approaches. Exosomes are nanoscale vesicles with diameters ranging from approximately 30–150 nm, capable of transporting a variety of molecules in the body and mediating intercellular communication. There is an increasing number of studies focusing on the role of exosomes in PF. This review demonstrates the significance of exosomes in the pathogenesis, diagnosis, and treatment of PF. Exosomes are able to influence inflammatory, immune, and extracellular matrix deposition processes in PF and regulate the corresponding cytokines. Some exosomes detected in sputum, blood, and bronchoalveolar lavage fluid may be used as potential diagnostic and prognostic biomarkers for PF. Exosomes derived from several cells, such as mesenchymal stem cells, have demonstrated potential as PF therapeutic agents. Drug delivery systems using exosomes may also provide new insights into PF therapy.

## 1 Introduction

Pulmonary fibrosis (PF) is an irreversible structural and functional change in the lung caused by excessive deposition of extra-alveolar matrix proteins resulting in fibrotic remodeling and alveolar destruction. PF is a severe risk to human life and health, with a median survival time of only 3–5 years after diagnosis of idiopathic pulmonary fibrosis (IPF). The pathogenesis of PF involves an inflammatory environment, oxidative stress, ageing, epigenetic and genetic and epithelial-mesenchymal transition (EMT) (Sundarakrishnan et al., 2018; Kinoshita and Goto, 2019; Yanagihara et al., 2020).

There are limited drugs available for the treatment of PF. Pirfenidone and nintedanib are commonly used in the treatment of PF. Although these two drugs can improve lung function and reduce clinical symptoms to some extent, they do not have significant advantages in improving survival and stopping or even reversing disease progression. And there are also uncertainties in the use of these drugs in terms of treatment duration, initial selection, etc., (Sgalla et al., 2018; Sgalla et al., 2020).

As exosomes have been refined in recent years, more and more studies have uncovered the significance of exosomes in the development of disease, and exosomes are expected to be a safe emerging method for the diagnosis and treatment of PF (Guiot et al., 2019; Xie and Zeng, 2020).

## 2 Exosomes

Exosomes are nanoscale vesicles ranging from approximately 30–150 nm in diameter, produced and released by most cells, and are a subtype of extracellular vesicles (EVs) (Yang D. et al., 2020). Exosomes can be found in body fluids such as blood, saliva, amniotic fluid, urine, semen, bronchial fluid, and cerebrospinal fluid (Doyle and Wang, 2019). The discovery of exosomes dates back to the 1980s when exosomes were first identified in reticulocytes (Harding et al., 1983; Pan et al., 1985; Johnstone et al., 1987), and were initially thought of simply as a means of removing unwanted cellular components (Johnstone, 1992). With the intensive study of exosomes, they are now considered to be involved in the regulation of intercellular communication, playing specific roles in biological processes such as immune response (Anel et al., 2019), antigen presentation (Arima et al., 2019), cell differentiation (Lloret-Llinares et al., 2018) and tumour invasion (Zhang and Yu, 2019; Rezaie et al., 2022).

### 2.1 Composition of exosomes

**FIGURE 1:**
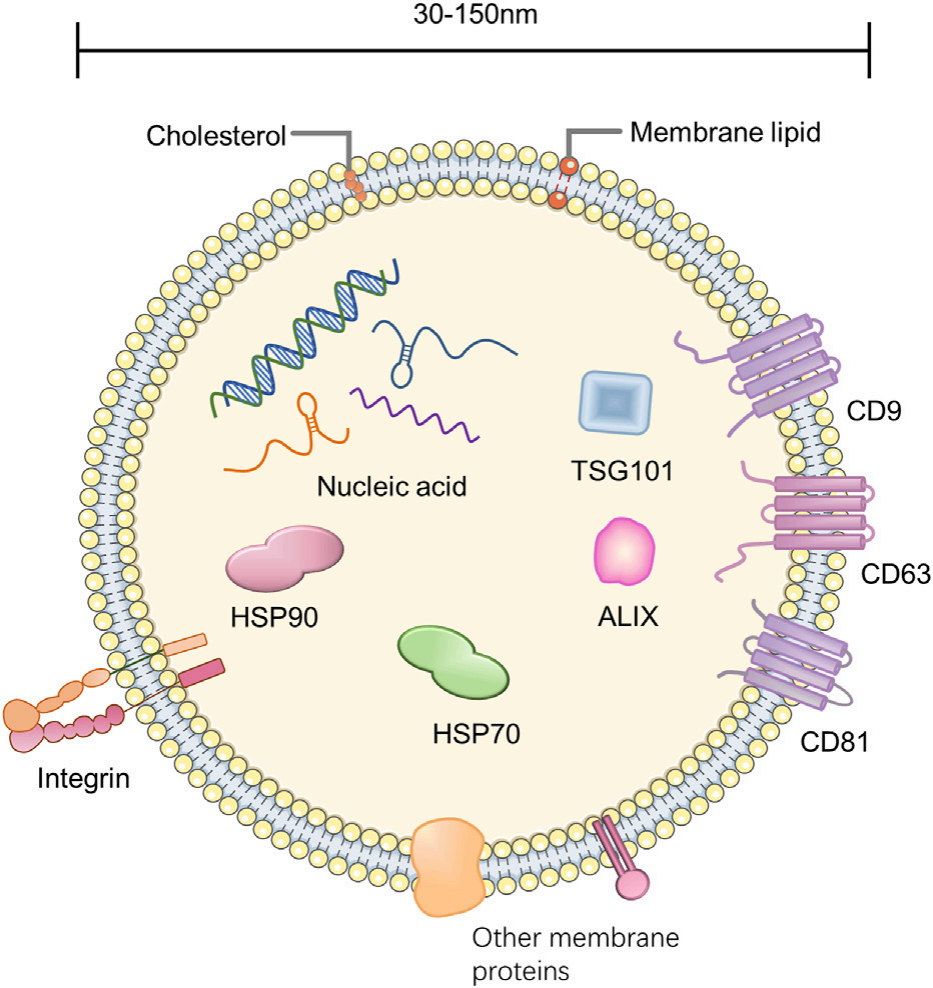
Composition of exosome.

The exosome is enclosed by a lipid bilayer and carries a variety of biologically active substances such as proteins, lipids, and nucleic acids (Wortzel et al., 2019) (Figure 1). The lipids of the exosome contain sphingolipids, phosphatidylserine, cholesterol, etc., (De Abreu et al., 2020). The nucleic acids carried by exosomes are divided into DNA and RNA, and RNA can include mRNA, microRNA (miRNA), tRNA, etc., (Keshtkar et al., 2021). Most exosomal proteins are associated with their formation and secretion, such as tetraspanins (e.g., CD9, CD63, CD81), heat shock proteins (e.g., HSP70, HSP90), TSG101, Alix, Rab GTPase, and Integrin. Furthermore, there are exosome-specific proteins secreted by specific cells, such as MHC-II from dendritic cells (Janockova et al., 2021; Liu et al., 2021).

### 2.2 Biogenesis, secretion and uptake of exosomes

**FIGURE 2:**
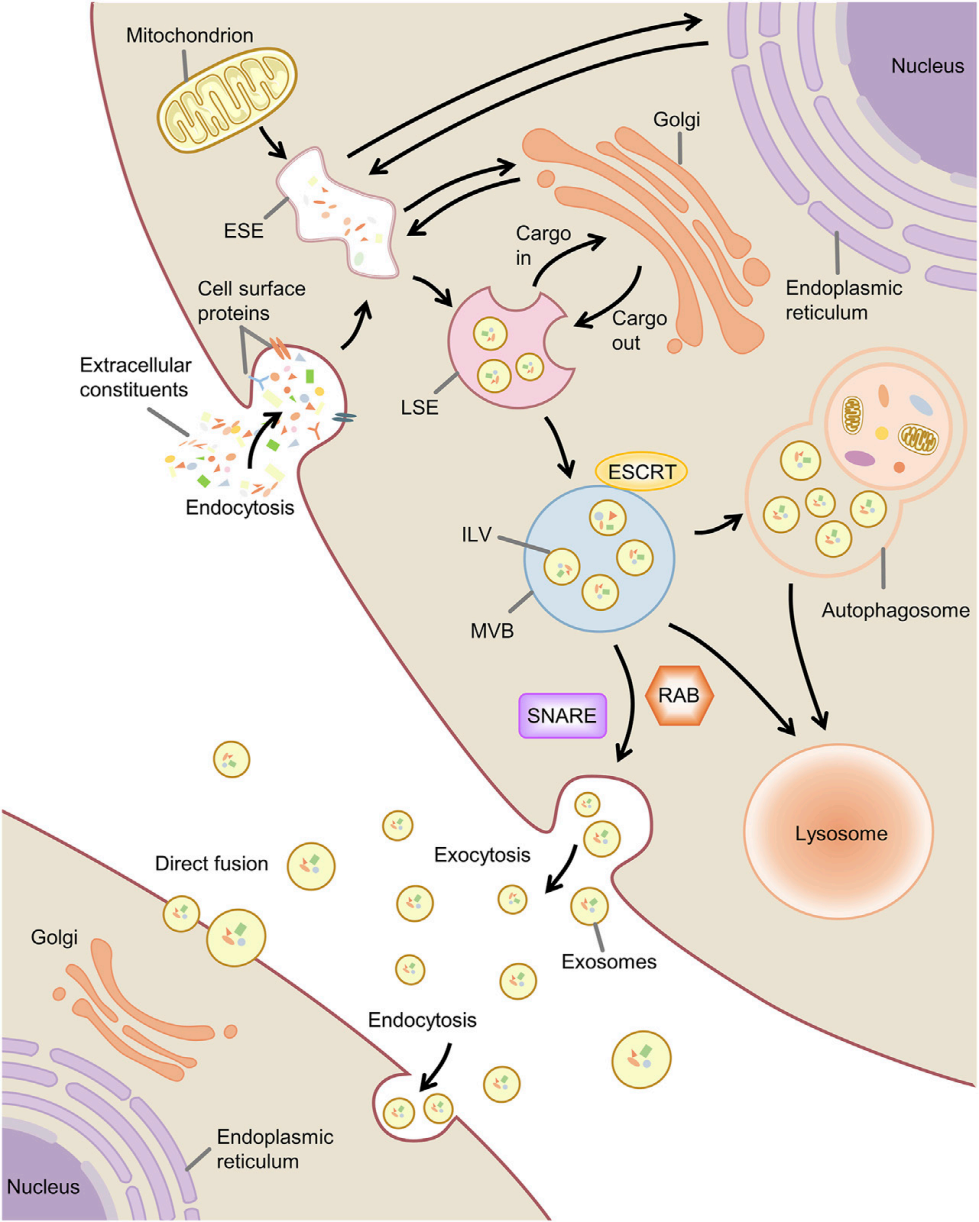
Biogenesis, secretion and uptake of exosome. Note: ESE, early-sorting endosome; LSE, late-sorting endosome; MVB, multivesicular body; ILV, intraluminal vesicle; ESCRT, endosomal sorting complex required for transport; SNARE, soluble N -ethylmaleimide-sensitive factor attachment protein receptor.

Exosome production is associated with the endosomal system. Early-sorting endosome (ESE) formation is caused by invagination of the plasma membrane and endocytosis of the extracellular component cell surface proteins (Kalluri and LeBleu, 2020). The endoplasmic reticulum, Golgi complex, and mitochondria can participate in the maturation of the ESE (Hoyer et al., 2018; Shi et al., 2018; Amari and Germain, 2021). ESE can mature into late-sorting endosome (LSE) and eventually produce multivesicular bodies (MVBs, also known as MVEs) (Hessvik and Llorente, 2018). During this process, the endosomal membrane invaginates to produce intraluminal vesicles (ILVs), and MVBs contain a large number of ILVs (Abels and Breakefield, 2016). A portion of MVBs can be degraded by autophagosomal or lysosomal fusion, and a portion of MVBs fuse with the plasma membrane to release the contained ILVs as exosomes to be taken up and function extracellularly (Hassanpour et al., 2020) (Figure 2).

There are currently thought to be two pathways that regulate the formation of ILVs, one involving the endosomal sorting complex required for transport (ESCRT) and the other independent of ESCRT (Osaki and Okada, 2019). ESCRT is mainly composed of four complexes (ESCRT-0, ESCRT-I, ESCRT-II, and ESCRT-III) and associated proteins (e.g., VPS4, ALIX, TSG101) (Larios et al., 2020). ESCRT plays a role in ubiquitin-mediated and ubiquitin-independent cargo recognition and sorting and can induce membrane deformation, with substrates of the ESCRT pathway ultimately available in translocated to ILVs (Frankel and Audhya, 2018).

Another pathway regulating the formation of ILVs involves mainly ceramide and tetraspanins. The exosomes contain ceramides, cholesterol, sphingomyelin, and tetraspanins (Toh et al., 2018). Ceramide is essential for exosome formation and secretion, but the exact mechanism is unclear. Ceramide has been found that may promote the inward budding of MVBs to produce ILVs by affecting membrane curvature (Elsherbini and Bieberich, 2018). Ceramides, cholesterol, and sphingolipids are involved in forming lipid rafts (Wei et al., 2021). Lipid rafts play an essential function in membrane budding, may contribute to the assembly of proteins or other molecules into endosomes, and can regulate the formation of epidermal growth factor receptor-containing ILVs (Li et al., 2018; Wei et al., 2021). Tetraspanins have been shown to regulate the transport of selectively associated proteins and their function through membrane compartmentalization and play a role in membrane fusion and the production of ILVs (Böker et al., 2018; Ghossoub et al., 2020).

Exosomes contain unique contents of nucleic acids, proteins, and lipids, and the sorting mechanism of these exosomal cargoes is closely related to the ILVs formation pathway described above. Exosomal miRNAs play an essential role in intercellular communication. The type and amount of miRNAs loaded into exosomes depend on the type of parental cells, cell-specific sorting mechanisms, and stimuli such as inflammation (Wozniak et al., 2020; Xie et al., 2022). According to current studies, the potential modes of miRNA sorting into exosomes can be summarized as the following: the neural sphingomyelinase 2-dependent pathway, the sumoylated heterogeneous nuclear ribonucleoproteins-dependent pathway, the 3′miRNA sequence-dependent pathway, and the miRNA-induced silencing complex-associated pathway. In addition, other membrane proteins (e.g., Vps4A), RNA-binding proteins (e.g., Argonaute 2, YB-1), and lipid rafts have also been associated with miRNA sorting (Wei H. et al., 2021; Xie et al., 2022).

The release of exosomes involves the translocation of MVBs to the plasma membrane and the fusion of MVBs with the plasma membrane. MVBs fuse with the plasma membrane and release the contained LIVs into the extracellular space as exosomes *via* exocytosis. The release of exosomes is currently thought to be associated with the cytoskeleton, Rab GTPase, and the soluble N-ethylmaleimide-sensitive factor attachment protein receptor (SNARE) (Hessvik and Llorente, 2018). The cytoskeleton regulates the translocation of MVBs to the plasma membrane, depending mainly on the interaction between actin and the microtubule cytoskeleton (Villarroya-Beltri et al., 2014; Gurunathan et al., 2021). Rab GTPases play a role in vesicle transport with the main functions of regulating vesicle budding, vesicle transport along with the cytoskeleton, and membrane fusion (Jin et al., 2021). For example, Rab11 facilitates the transportation of MVBs and the fusion of MVBs with the plasma membrane (Savina et al., 2005; Messenger et al., 2018), and Rab27 can regulate the docking of MVBs with the plasma membrane (Song et al., 2019), and Rab35 is a critical factor in regulating the transport of MVBs (Yang et al., 2019). SNARE is a central mechanism that mediates membrane fusion (Wang et al., 2017). It has been found to facilitate the docking of MVBs to the plasma membrane and thus contribute to exosome release (Yu et al., 2020).

After releasing outside the cell, exosomes are taken up and acted upon by the recipient cells mainly through membrane fusion and endocytosis (Gonda et al., 2019). The exosome uptake mechanism largely depends on the recipient cell (Horibe et al., 2018).

## 3 The function of exosomes in PF

**FIGURE 3:**
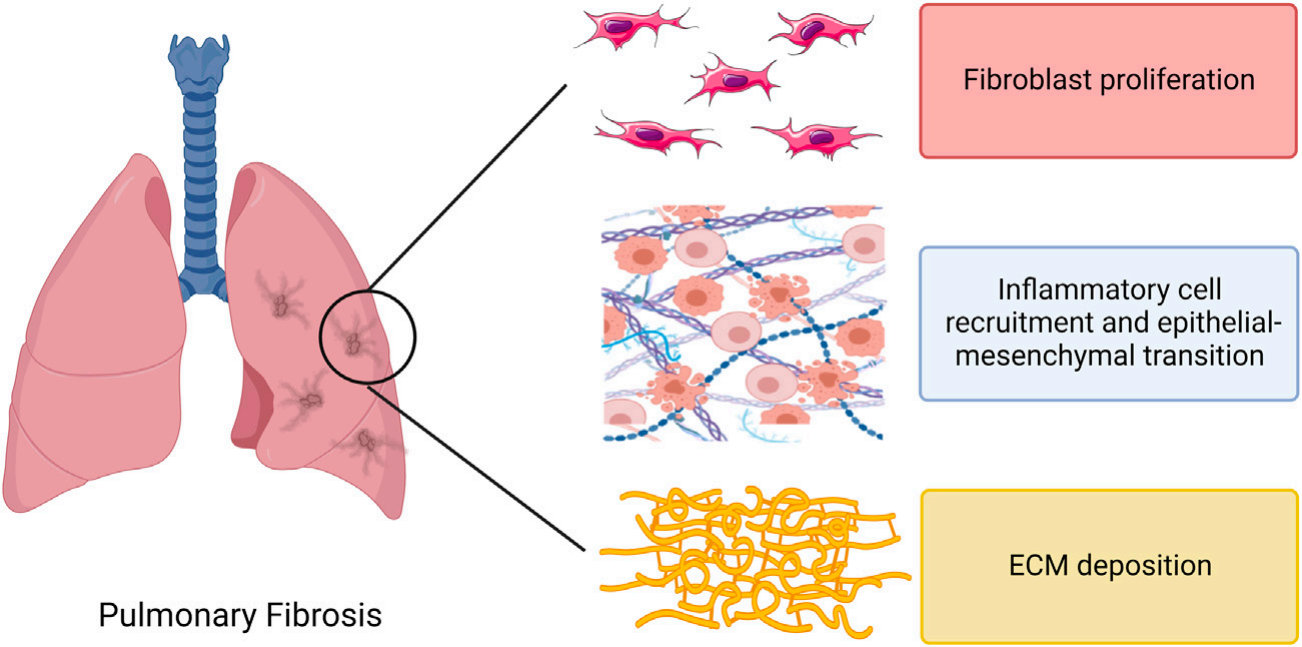
Direct effects of regulatory immune cells, fibroblasts and extracellular matrix in pulmonary fibrosis. Note: ECM, extracellular matrix.

**FIGURE 4:**
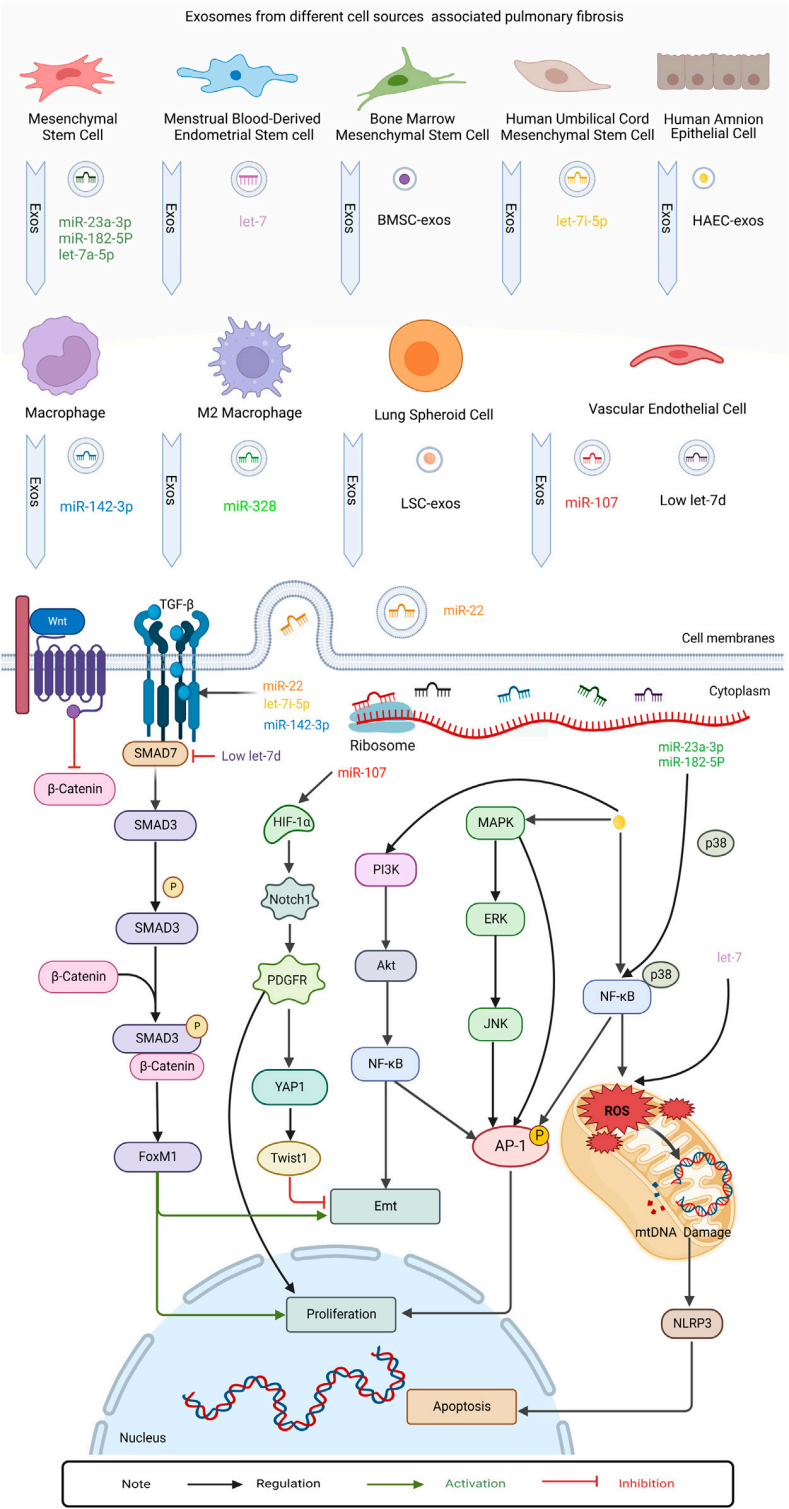
This figure summarizes the role of regulatory exosomes in PF. Mechanism investigation revealed that exosomes secreted by different kinds of cells, eg: human amnion epithelial cell (HAEC), Menstrual Blood-Derived Endometrial Stem Cell (MenSC), Mesenchymal stem cells (MSC), Human umbilical cord mesenchymal stem cell (HUCMSC), Bone marrow mesenchymal stem cell (BMSC), Pluripotent stem cell (iPSC), Lung spheroid cell(LSC), Vascular endothelial cell (EnC), Macrophage, M2 macrophages. The exosomes transmitted protein cargo and microRNAs regulated MAPK, NF-kb, PI3k/Akt, Wnt/β-catenin, TGFβRI/FoxM1/Smad/β-catenin, HIF-1α/Notch1/PDGFRβ/YAP1/ Twist1 signaling pathways. Finally these pathways assess their interaction with epithelial-mesenchymal transition, apoptosis and proliferation in contradicting findings.

In recent years, an increasing number of studies have focused on the role played by exosomes in PF. The initiation of PF is associated with lung injury and impaired repair, with multiple causes of lung epithelial cell injury followed by inflammatory cell recruitment and massive pro-inflammatory cytokine release, triggering an immune response. Monocytes and macrophages can drive fibrosis through an over-repair response to lung injury. The pro-fibrotic mediators act on lung fibroblasts, causing excessive proliferation and differentiation of fibroblasts, which in turn promotes the proliferation of myofibroblasts and the production of extracellular matrix (ECM), ultimately leading to PF pathological features such as excessive ECM deposition in lung tissue (Mei et al., 2021) (Figure 3). It has been reported in several publications that exosomes can influence inflammation and immunity, fibroblast proliferation and differentiation, ECM deposition, and other aspects of the PF disease process. The functions of exosomes reported so far in PF are highlighted in Figure 4 and discussed in more detail in subsequent sections.

### 3.1 Exosomes affect inflammation and immunity in PF

Fibrosis is the result of excessive trauma repair and tissue remodeling, which is considered to be chronic inflammation and eventual fibrosis caused by epithelial injury (Heukels et al., 2019). Inflammatory stimulation or persistence of the inflammatory state is an essential condition for the development of PF. Following injury, damaged epithelium and recruited inflammatory cells secrete a variety of mediators, including transforming growth factor-β (TGF-β) to regulate epithelial repair and promote fibroblast recruitment and myofibroblast activation (Hewlett et al., 2018).

Macrophages can polarize to M1 and M2 phenotypes after different environmental and cytokine stimuli. Under chronic inflammatory conditions, pro-inflammatory M1 macrophages slowly convert to the more anti-inflammatory M2 phenotype, secreting mediators that promote wound healing. M2-type macrophages regulate fibrosis by secreting various growth factors (including TGF-β) and other mechanisms (Heukels et al., 2019).

Oxidative stress and inflammation are closely linked and play a pro-fibrotic role in the fibrotic process. Under sustained stimulation, abnormally functioning mitochondria overproduce reactive oxygen species (ROS) (Lv et al., 2019). ROS can be involved in inflammation by affecting NF-κB signaling, TNF signaling, and so on (Blaser et al., 2016). In addition, abnormal production and accumulation of ROS can lead to cellular damage at the level of nucleic acids, proteins, lipids, and carbohydrates, thus participating in the pathogenesis of PF (Cameli et al., 2020).

Exosomes can down-regulate inflammatory cell infiltration during PF disease and participate in the regulation of PF disease through the mediation of some classical inflammatory pathways such as NF-κB, MAPK, and other pathways. In these studies, miRNAs contained in exosomes such as miR-23a-3p and miR-182-5p could play an important role in the regulation of PF (Tan et al., 2018; Royce et al., 2019; Xiao et al., 2020; Kaur et al., 2021). Exosomes can also regulate PF by affecting ROS. For example, exosomal let-7 exerts an anti-PF effect by regulating ROS and mitochondrial DNA damage (Sun et al., 2019).

Some studies have focused on the effect of exosomes on the differentiation and proliferation of immune cells. In these studies, exosomes could alleviate PF by modulating the phenotypic switch of monocytes and macrophages, enhancing macrophage phagocytosis, and inhibiting T cell proliferation (Tan et al., 2018; Mansouri et al., 2019; Zhou et al., 2021). For example, exosomes from induced pluripotent stem cells (iPSCs) attenuate PF by suppressing M2-type macrophages during PF through the miR-302a-3p/TET1 axis (Zhou et al., 2021).

Macrophages can participate in the progression of PF through their secreted exosomes. Macrophage-derived exosomes promote differentiation, proliferation, and migration of myofibroblasts after exposure to silica (Qin et al., 2021). In bleomycin-induced PF, one study found that macrophage-derived exosomes mediated intercellular communication between macrophages and fibroblasts by transferring angiotensin II type 1 receptors to lung fibroblasts (Sun N. N. et al., 2021). Another study showed that the M2 macrophage-derived exosome miRNA-328 enhances lung interstitial fibroblast proliferation and promotes PF by regulating FAM13A (Yao et al., 2019).

### 3.2 Exosomes affect ECM deposition in PF

Excessive ECM deposition is a characteristic feature of PF. The large number of cytokines produced after epithelial injury in PF stimulates fibroblasts to proliferate and differentiate into myofibroblasts, which leads to the production of high amounts of collagen, fibronectin, and other ECM proteins and their deposition in the interstitial lung, leading to reduced gas exchange and impaired lung function, and ultimately to respiratory failure (Chanda et al., 2019; Bueno et al., 2020; Mei et al., 2021).

A study showed that exosomes in bronchoalveolar lavage fluid (BALF) of PF rats, specifically exosome miR-204-5p, promote fibroblast proliferation and collagen deposition in lung tissue, indicating that exosomes may participate in the PF process by regulating ECM deposition (Zhu et al., 2021). Long noncoding RNA (lncRNA) HOTAIRM1 delivered by alveolar epithelial cell-derived exosomes was found to play a role in PF, and lncRNA HOTAIRM1 was able to promote lung fibroblast proliferation and differentiation and contribute to ECM remodeling by regulating the miR-30d-3p/HSF1/YY1 axis (Chen et al., 2022). Zhang et al. (2021) found that exosomes derived from bone marrow mesenchymal stem cells in a silicosis PF model reduced collagen accumulation in lung tissue and inhibited the expression of components of the Wnt/β-catenin pathway, which is closely related to EMT. Wnt5a was also found to be directly transported by EVs in PF and induced fibroblast proliferation, which incidentally consisted mainly of exosomes in that study (Martin-Medina et al., 2018). Phuong-Uyen C. Dinh et al. (2020) found that lung spheroid cell-derived exosomes (LSC-EXOs) reduced collagen deposition in PF induced by bleomycin or silica, in addition to inhibiting the proliferation of myofibroblasts. The above study illustrates the role of exosomes in regulating the proliferation of fibroblasts and myofibroblasts, which are the primary sources of ECM in PF, and this may be an entry point to investigate the mechanisms by which exosomes regulate ECM deposition (Tsukui et al., 2020).

### 3.3 Exosomes regulate PF-related cytokines

Cytokines can be involved in multiple aspects of inflammation and ECM deposition in PF (Luzina et al., 2015). TGF-β plays a vital role in the progression of PF, mediating fibroblast recruitment and differentiation, promoting ECM deposition, and inducing EMT. TGF-β can exert pro-fibrotic effects in cooperation with platelet-derived growth factor (PDGF), which itself can be involved in lung fibroblast proliferation and collagen synthesis (Chanda et al., 2019; Wang et al., 2020). Other cytokines such as connective tissue growth factor (CTGF), insulin-like growth factor, and CCL2 also modulate PF (Luzina et al., 2015).

Several studies have shown that exosomes can regulate PF through cytokines such as TGF-β. Activated TGF-β binds to its receptor and regulates the expression of target genes, including pro-fibrotic genes, through Smad proteins (Massagué, 2012). Smad7 is a negative regulator of TGF-β signaling that blocks the TGF-β1/Smads signaling pathway and inhibits fibroblast differentiation. A study elucidated that in cigarette smoke-induced PF, exosomes promote abnormal epithelial-fibroblast crosstalk by inhibiting Smad7 to activate the TGF-β1/Smad3 pathway (Bai et al., 2021). TGF-β1 and its receptor can regulate the transformation of lung pericytes into myofibroblasts. Han Xie et al. found that the lung vascular endothelium-derived exosome low let-7d targets transforming growth factor β receptor 1 (TGFβR1) to regulate the TGFβRI/FoxM1/Smad/β-catenin pathway, which drives lung pericytes fibrosis (Xie et al., 2020). In addition, exosomal miR-22 was found to significantly inhibit TGF-β1-induced α-smooth muscle actin (α-SMA) expression (Kuse et al., 2020). Guiot et al. (2020) found that the macrophage-derived exosome miR-142-3p was able to reduce TGFβR1 expression and inhibit TGF-β-induced fibrotic responses in alveolar epithelial cells and lung fibroblasts by targeting TGFβR1. The finding of another study demonstrated that human umbilical cord mesenchymal stem cell-derived exosome let-7i-5p could target TGFβR1 and inhibit lung fibroblast activation via the TGFβR1/Smad3 signaling pathway in a silica-induced PF model (Xu et al., 2022).

Platelet-derived growth factor receptor β (PDGFRβ) is expressed in pericytes, and a study demonstrated that the pulmonary vascular endothelial cell-derived exosome miR-107 could target HIF-1α to regulate PDGFRβ and mitigate pericyte-promoted PF progression by inhibiting the HIF-1α/Notch1/PDGFRβ/YAP1/Twist1 signaling pathway (Wang et al., 2021). Kuse et al. (2020) found in an *in vitro* PF model that exosomal miR-22 could attenuate the expression of CTGF, which is an important mediator of fibrosis and stimulates lung fibroblast differentiation, thereby inhibiting CTGF-induced α-SMA expression.

## 4 Exosomes as biomarkers of PF

The current diagnosis of PF is not straightforward and requires multidisciplinary collaboration between pulmonologists, pathologists, and radiologists, and there remains a recognized need for an accurate, early diagnosis of PF. Early diagnosis of PF is critical for prognosis with survival being poor in several fibrotic interstitial lung diseases, including IPF, and patients with PF may benefit more from early diagnosis and timely disease management (Spagnolo et al., 2012; Cottin et al., 2018). Biomarkers have objective detection and evaluation properties and can be used as indicators of physiological/pathological processes or pharmacological responses to therapeutic interventions, which are essential in PF (Guiot et al., 2017). To date, several studies have shown that exosomes detected in body fluids such as blood and sputum have the potential to be diagnostic, prognostic, and predictive biomarkers of PF (Table 1).

**TABLE 1:** Biomarker potential of exosomes in PF.

Exosome—Cargos	Expression in PF (vs. Controls)	Source	Cohort selection	Clinical implication	References
let-7d	Downregulated	Serum	61 IPF patients and 15 controls	Promising diagnostic biomarkers for IPF	Lacedonia et al. (2021)
miR-16	Downregulated				
miR-142-3p	Upregulated	Sputum	16 IPF patients and 14 controls	Potential biomarkers for diagnosis and prediction of disease severity in IPF	Njock et al. (2019)
miR-33a-5p	Upregulated				
let-7d-5p	Downregulated				
miR-142-3p	Upregulated	Sputum	19 IPF patients and 23 controls	Promising diagnostic biomarkers for IPF	Guiot et al. (2020)
		Plasma	14 IPF patients and 14 controls		
miR-22-3p	Upregulated	BALF	8 IPF patients and 8 controls	Promising diagnostic biomarkers for IPF	Kaur et al. (2021)
miR-320a-3p	Upregulated				
miR-320b	Upregulated				
miR-24-3p	Upregulated				
miR-375-3p	Downregulated				
miR-200a-3p	Downregulated				
miR-200b-3p	Downregulated				
miR-141-3p	Downregulated				
miR-423-5p	Downregulated				
miR-125b	Upregulated	BALF	30 IPF patients and 16 controls	Promising diagnostic biomarkers for IPF	Liu et al. (2018)
miR-128	Upregulated				
miR-21	Upregulated				
miR-100	Upregulated				
miR-140-3p	Upregulated				
miR-374b	Upregulated				
let-7d	Downregulated				
miR-103	Downregulated				
miR-26	Downregulated				
miR-30a-5p	Downregulated				
miR-21-5p	Upregulated	Serum		Potential biomarkers for diagnosis and prognosis of IPF	Makiguchi et al. (2016)
CD19	Upregulated	Serum	90 IPF patients and 19 controls	Potential biomarkers for diagnosis of IPF; CD8 is a potential prognostic biomarker for IPF	D'Alessandro et al. (2021)
CD69	Upregulated				
CD8	Upregulated				
CD86	Upregulated				
CD209	Upregulated				
Cd133/1	Upregulated				
MCSP	Upregulated				
ROR1	Upregulated				
CD42a	Downregulated				

Note: PF, pulmonary fibrosis; IPF, idiopathic pulmonary fibrosis; BALF, bronchoalveolar lavage fluid.

Hematological tests, sputum examination, and bronchoscopy are diagnostic tools for respiratory diseases, and exosomal miRNAs can be detected by these tests. Several studies have reported the potential of some exosomal miRNAs as biomarkers for the diagnosis of PF. Donato Lacedonia et al. (2021) analyzed the expression of exosomal miRNAs in the serum of IPF patients and found that let-7d and miR-16 were down-regulated compared to controls. Previous studies have shown that let-7d has a key regulatory role in the prevention of PF, while fewer works have been conducted on the association between miR-16 and PF, and further studies are necessary to determine their ability to act as biomarkers of IPF (Pandit et al., 2010; Huleihel et al., 2014; Liang et al., 2016; Min et al., 2016). Makon-Sébastien Njock and his colleagues worked on finding exosomal biomarkers of IPF in sputum. By analyzing sputum samples from IPF patients and healthy controls, they found that the significantly upregulated exosomes miR-142-3p, miR-33a-5p, and downregulated exosome let-7d-5p in sputum were promising diagnostic biomarkers, and notably, they demonstrated that exosomal miR-142-3p was negatively correlated with diffusing capacity/alveolar volume of carbon monoxide and exosomal let-7d-5p was positively correlated, which may help to evaluate the severity of IPF (Njock et al., 2019). When they further investigated the relationship between exosomal miR-142-3p and IPF, they found that in addition to its potential as a sputum biomarker for IPF, exosomal miR-142-3p was significantly upregulated in the plasma of IPF patients, making it more promising as a diagnostic modality for IPF (Guiot et al., 2020). Kaur et al. (2021) compared IPF patients with healthy nonsmokers and detected 55 differentially expressed exosomal miRNAs in IPF human lung tissue, also finding significant downregulation of exosomes miR-375-3p, miR-200a-3p, miR-200b-3p, miR-141-3p, and miR-423-5p and upregulation of exosomes miR-22-3p, miR-320a-3p, miR-320b, and miR-24-3p in BALF-derived exosomes in IPF patients. Bronchoscopic lung cryobiopsy is a promising examination technique in the diagnosis of PF, allowing safer and more effective access to lung tissue with BALF, thus providing a source of feasibility for BALF exosomal miRNAs to become a diagnostic tool for PF (Tomassetti et al., 2016). Another study showed that exosomes miR-125b, miR-128, miR-21, miR-100, miR-140-3p, and miR-374b were upregulated in BALF from IPF patients, while let-7d, miR-103, miR-26, and miR-30a-5p were downregulated (Liu et al., 2018). These findings suggest the potential of BALF-derived exosomal miRNAs as PF biomarkers.

Furthermore, Makiguchi et al. (2016) found that serum EV miR-21-5p was upregulated throughout the progression of PF compared to controls in an animal model of bleomycin-induced PF, and they observed significantly higher EV miR-21-5p in the sera of IPF patients compared to healthy controls, suggesting that EV miR-21-5p is a candidate biomarker for IPF. Tomonori Makiguchi and his colleagues also confirmed the potential of serum EV miR-21-5p as a prognostic biomarker for IPF. According to their study, serum EV miR-21-5p correlated with the rate of lung volume loss in IPF patients, and Kaplan-Meier analysis indicated that patients with higher baseline serum EV miR-21-5p levels had significantly lower survival rates. Given that the investigators obtained serum EV miR-21-5p by Total Exosome Isolation reagent, serum exosome miR-21-5p may have the same biomarker potential. Kaur et al. (2020) suggested that downregulation of an exosomal lncRNA encoding a WT1 transcription factor in the plasma of smokers may represent a possibility for them to develop PF in the future . Miriana d'Alessandro et al. examined blood exosome surface epitopes in IPF patients and found many exosome surface epitopes CD19, CD69, CD8, CD86, CD209, Cd133/1, MCSP, and ROR1 were significantly higher in IPF patients and CD42a was lower than in controls. In addition, increased expression of CD8 was associated with poor prognosis in IPF in survival analysis. These results provide the basis for the use of blood exosome surface epitopes as candidate biomarkers for the diagnosis and prognosis of IPF (D'Alessandro et al., 2021).

## 5 Exosomes in PF treatment

### 5.1 Potential of exosomes as PF therapeutics

Lung transplantation is currently the most effective treatment for most PF. Still, only a small number of patients can benefit from it due to the limited availability of donor organs, rejection reactions, and the cost of treatment. The drug treatment options for PF are relatively limited, and taking IPF as an example, the main treatment options for IPF are nintedanib, pirfenidone, and a combination of both. Although they can slow down the disease progression of PF to some extent, these therapies have adverse drug reactions and do not have the ability to cure the disease. Many patients with PF have a low quality of life and a short survival time after diagnosis. Thus there is still an urgent need for a treatment that can stop or even reverse PF. An increasing number of studies are currently dedicated to the search for safe and effective new therapies for PF (Richeldi et al., 2017; Mei et al., 2021). As previously described, exosomes have the ability to regulate fibroblast proliferation and differentiation, ECM deposition, and EMT, and several studies have demonstrated the therapeutic potential of exosomes for PF in animal models, as shown in Table 2, suggesting that exosomes may be a valuable clue to address this issue of developing new therapies for PF.

**TABLE 2:** Potential therapeutic implication of exosomes in PF.

Source of exosomes	Extraction method	Experimental model	Exosome - cargos	Species	Delivery of exosomes	Potential therapeutic implication	References
BMSC	Differential Ultracentrifugation	Silca	Unknown	Rats	Intravenous injection	Alleviate PF, reduce collagen accumulation, inhibit TGF-β1 and decrease HYP content	Zhang et al. (2021)
BMSC	iodixanol (OptiPrep) cushion density flotation	Bleomycin	Unknown	Mice	Intravenous injection	Alleviate PF, reduce collagen accumulation and reduce the degree of apoptosis	Mansouri et al. (2019)
hucMSC	Ultracentrifugation + Exoquick exosome precipitation solution	Silca	Unknown	Mice	Intravenous injection	Alleviate PF, improve respiratory impairment and reduce collagen deposition in NIH-3T3 cells	Xu et al. (2020)
hucMSC	Ultracentrifugation + Exoquick exosome precipitation solution	Silca	let-7i-5p	Mice	Intravenous injection	Affect the expression of α-SMA, collagen type I, fibronectin, TGFβR1 and P-Smad3 in NIH-3T3 cells, and inhibit fibroblast activation	Xu et al. (2022)
hucMSC	Differential centrifugation	Bleomycin	Unknown	Mice	Intravenous injection	Reduced lung coefficient, improved lung histopathological changes and collagen deposition in mice with PF, reduced TGF-β1 expression, inhibited p-Smad2/3 and vimentin expression and increased E-cadherin expression	Yang et al. (2020b)
MSC	Ultracentrifugation	Lipopolysaccharide	miR-23a-3p miR-182-5p	Mice	Intravenous injection	Alleviate PF and injury, attenuate apoptosis and EMT progression	Xiao et al. (2020)
MSC	Ultracentrifugation	Monocrotaline	Unknown	Rats	Intravenous injection	Attenuate PF and pulmonary vascular remodelling, reduce right ventricular systolic pressure and right ventricular hypertrophy index	Zhang et al. (2020b)
MSC	Differential Centrifugation + Iodixanol (OptiPrep)	Hyperoxia	Unknown	Mice	Intravenous injection	Alleviate PF, reduce collagen accumulation, reduce pulmonary vascular remodelling and inflammation, improve lung function	Willis et al. (2018)
iPSC	Ultracentrifugation	Bleomycin	miR-302a-3p	Mice	Intravenous injection	Relieve PF, reduce collagen deposition, and inhibit the increase of M2 type macrophages	Zhou et al. (2021)
hESC	Ultracentrifugation	Radiation	Unknown	Mice	Intravenous injection	Alleviates PF and reduces collagen accumulation, macrophage infiltration and inflammation	Montay-Gruel et al. (2020)
MenSC	Differential centrifugation + exosome extraction kit (Wako Pure Chemicals Industry)	Bleomycin	let-7	Mice	Intravenous injection	Alleviate PF and alveolar epithelial cell damage, reduce collagen accumulation in lung tissue and adjust wet and dry specific gravity	Sun et al. (2019)
hAEC	Ultracentrifugation	Bleomycin	Unknown	Mice	Intranasal administration	Alleviate PF, reduce collagen accumulation and lung inflammation	Tan et al. (2018)
hAEC	Ultracentrifugation	Bleomycin	Unknown	Mice	Intranasal administration	Alleviate PF, reduce airway remodeling and inflammation, with the above effects being better when exosomes are combined with Serelaxin	Royce et al. (2019)
LSC	Ultrafiltration + Centrifugation	Bleomycin	Unknown	Mice	Nebulised inhalation	Alleviate PF, restore normal alveolar structure, reduce collagen accumulation and myofibroblast proliferation, and improve lung function	Dinh et al. (2020)
		Silica		Rats			
—	—	Bleomycin	miR-16	Mice	Intravenous injection	Relieve PF and reduce collagen accumulation	Inomata et al. (2021)
—	—	Bleomycin	miR-22	Mice	Intravenous injection	Alleviate PF, reduce PF scores and collagen accumulation	Kuse et al. (2020)

Note: BMSC, bone marrow mesenchymal stem/stromal cell; hucMSC, human umbilical cord mesenchymal stem cell; MSC, mesenchymal stem/stromal cell; iPSC, induced pluripotent stem cell; hESC, human embryonic stem cell; hAEC, human amnion epithelial cell; MenSC, menstrual blood-derived endometrial stem cell; LSC, lung spheroid cell; EnC, vascular endothelial cell; α-SMA, α-smooth muscle actin; TGF-β:transforming growth factor-β; TGFβR1, TGF-β receptor 1; P-Smad: phosphorylated smad; —, no relevant data.

Mesenchymal stem/stromal cell-derived exosomes (MSC-EXOs) have been proposed as promising therapeutic tools for a variety of diseases (Ahmadi and Rezaie, 2021; Janockova et al., 2021) and have shown anti-fibrotic potential such as for liver, kidney, and cardiac fibrosis (Li et al., 2013; Eirin et al., 2017; Rong et al., 2019; Chen et al., 2020). MSCs are also a preferred source of exosomes for the study of PF treatment. Mansouri et al. (2019) found that bone marrow mesenchymal stem/stromal cell-derived exosomes (BMSC-EXOs) were effective in attenuating bleomycin-induced PF and modulating lung inflammation, and interestingly, the investigators observed that BMSC-EXOs significantly restored bleomycin-induced PF in mice after intravenous injection of BMSC-EXOs on day 7 post-modeling. The ability of BMSC-EXOs to reverse PF is of interest and needs to be studied in depth. In addition to this, they found that bone marrow-derived mononuclear cell transplantation pretreated by BMSC-EXOs could exert a preventive effect on PF. Another study demonstrated the ability of BMSC-EXOs to effectively alleviate silica-induced PF in a rat model of silicosis (Zhang et al., 2021). There are no effective therapies to slow down the progression of silicosis, especially its fibrosis, and exosomes offer a therapeutic idea for PF in silicosis. Xu et al. (2020) found that human umbilical cord MSC-derived exosomes (hucMSC-EXOs) exerted anti-PF effects in a mouse silicosis model, effectively reducing collagen deposition, attenuating pulmonary fibrosis-related indices, and improving impaired lung function in silicosis mice. Chunjie Xu and colleagues found through a more in-depth study that the exosome let-7i-5p in hucMSC-EXOs effectively attenuated collagen deposition in silicosis mice and improved silica-induced PF in mice by inhibiting fibroblasts (Xu et al., 2022). Another study elucidated that hucMSC-EXOs had the effect of alleviating bleomycin-induced PF and significantly attenuated the pathological changes, and reduced collagen deposition in the lung tissue of PF mice (Yang J. et al., 2020). Acute lung injury can lead to fibrosis in lung tissue. In studying the mechanisms by which MSC regulates the progression of acute lung injury, Xiao et al. (2020) found that MSC-EXOs have the potential to attenuate lipopolysaccharide-induced PF progression, and the investigators demonstrated that miR-23a-3p and miR-182-5p transported by MSC-EXOs have the ability to reverse the EMT process that is closely associated with PF. Zhang Z. et al. (2020b) found that MSC-EXOs also had the ability to attenuate PF in a rat model of monocrotaline-induced pulmonary arterial hypertension. Another study indicated that MSC-EXOs could also attenuate PF and improve lung function in a mouse model of hyperoxia-induced bronchopulmonary dysplasia (Willis et al., 2018).

Stem cell-derived exosomes other than MSCs have also demonstrated promising therapeutic effects on PF in several studies. In view of the potential therapeutic value of iPSCs and their derivatives in several diseases, Yan Zhou and colleagues, after observing the potential of iPSCs for the treatment of PF, further built on their findings that iPSC-derived exosomes (iPSC-EXOs) could alleviate bleomycin-induced PF in mice, and that miR-302a-3p carried by iPSC-EXOs was able to reduce collagen deposition and inhibit M2-type macrophage increase in mouse lungs (Zhou et al., 2016; Zhou et al., 2021). Pierre Montay-Gruel et al. administered human embryonic stem cell (hESC)-derived EVs intravenously to mice receiving chest radiation exposure, most of which were actually exosomes, and observed a significant reduction in radiation-induced PF. This study suggests that hESC-derived exosomes may have the potential to become a therapeutic pathway for radiation therapy-induced PF (Montay-Gruel et al., 2020). Menstrual blood-derived endometrial stem cells (MenSCs) have antifibrotic effects, and Lifang Sun et al. found that MenSC-derived exosomes (MenSC-EXOs) ameliorated bleomycin-induced PF and alveolar epithelial cell injury in mice, which may be closely related to exosomal let-7. The investigators observed that let-7 carried by MenSC-EXOs could inhibit the process of EMT in mice and attenuate lung histopathological changes and that MenSC-EXOs could also translocate let-7 into alveolar epithelial cells to attenuate cell damage (Sun et al., 2019).

In addition to the stem cell-derived exosomes described above, several other types of exosomes have the potential for the treatment of PF. Tan et al. (2018) endeavored to explore the feasibility of human amnion epithelial cell-derived exosomes (hAEC-EXOs) as a potential therapy for IPF. They performed early and late interventions in juvenile mice with bleomycin-induced PF via intranasal drip of hAEC-EXOs and found that hAEC-EXOs were able to exert anti-inflammatory and anti-fibrotic effects at different stages of PF, with the potential to prevent, mitigate and even reverse PF. They also showed that the aforementioned anti-PF effects of hAEC-EXOs in juvenile mice were reproduced in aged mice, which were used to simulate the clinical setting of elderly patients with IPF, demonstrating the therapeutic potential of hAEC-EXOs for various disease stages and ages of PF. Another study found that hAEC-EXOs, in combination with the anti-fibrotic drug serelaxin, exerted better anti-inflammatory, anti-fibrotic, and alleviating airway dysfunction in bleomycin-induced PF mice (Royce et al., 2019). Dinh et al. (2020) used a nebulizer to treat bleomycin-induced and silica-induced PF mice with nebulized inhaled lung spheroid cell exosomes (LSC-EXOs) and found that LSC-EXOs exerted anti-PF effects in both PF mouse models, reducing collagen accumulation and myofibroblast proliferation, and improving impaired lung function. In addition, their results suggest that LSC-EXOs may be superior to MSC-EXOs in attenuating PF. Exosomal miR-107 and let-7d produced by pulmonary vascular endothelial cells have been reported to inhibit the function of pericytes in PF, which provides a promising therapeutic target for PF (Xie et al., 2020; Wang et al., 2021). In addition, exosomal miR-22 and exosomal miR-16 demonstrated experimentally their ability to ameliorate bleomycin-induced PF in mice through their mimics (Kuse et al., 2020; Inomata et al., 2021). In most of the above studies, an intravenous injection was chosen as the mode of exosome administration. Interestingly, in exploring the therapeutic potential of hAEC-EXOs and LSC-EXOs for PF, the investigators chose intranasal drip and nebulized inhalation administration, respectively, which enriches the effective routes of administration in anti-PF exosomes.

### 5.2 Potential of exosomes as PF drug delivery vehicles

With the development of nanomedicine, more and more literature has reported that nano-drug delivery systems can function as stable, safe, and effective drug delivery, and the progressive exploration of pulmonary drug delivery vehicles, including exosomes, has helped to advance the development of PF therapy (Skibba et al., 2020). Compared to nano-drug delivery systems such as *in vitro* synthesized liposomes, exosomes have unique drug delivery advantages. Exosomes are naturally secreted by organismal cells, can be widely and stably present in body fluids, and have better biocompatibility and lower immunogenicity. The lipid-rich composition of exosomes facilitates their loading of hydrophilic and lipophilic active pharmaceutical ingredients, which happens to be lipophilic for pirfenidone, a therapeutic drug for PF (Haak et al., 2017; Guiot et al., 2019). Exosomes are capable of mediating intercellular communication in the body, have the ability to target tissues and cells, and can cross biological barriers such as the blood-brain barrier (Zhang Y. et al., 2020; Saint-Pol et al., 2020). With these properties and abilities, exosomes have the potential to deliver PF therapeutic agents such as drugs and miRNAs.

There are already studies invested in the development of exosomes as drug delivery systems for anti-PF agents. Lingna Sun and coworkers designed a hybrid drug delivery system of fibroblast-derived exosomes and clodronate (CLD)-loaded liposomes (EL-CLD), which utilizes the affinity of fibroblast-derived exosomes for fibroblasts and CLD to reduce the uptake of drugs by the liver, proposing to solve the problem of poor selectivity and low delivery efficiency of PF therapeutic drugs to fibroblasts. In an animal model of PF, the investigators found that the anti-PF efficacy of Nintedanib was significantly increased when it was loaded with EL-CLD, thanks to the ability of EL-CLD to increase the delivery of nintedanib to the lung and its accumulation in fibrotic lung tissue, as well as to attenuate the macrophage-induced inflammatory response (Sun L. et al., 2021). Notably, the team has also developed another hybrid exosome-liposome drug delivery system that enhances the efficacy of nintedanib in the treatment of liver fibrosis (Ji et al., 2022). These findings highlight that the ability of exosomes to target delivery enables them to be advantageous in drug delivery, and furthermore, they demonstrate the feasibility and effectiveness of exosomes as delivery vehicles for anti-fibrotic, especially anti-PF drugs. However, the technology of exosome application for drug delivery is still immature. Problems such as how to extract exosomes efficiently and completely, how to improve the drug loading rate of exosomes, how to surface modify exosomes to improve their stability, and the types of drugs delivered are limiting the further development of this technology, and more research needs to be invested in tackling these challenges (Zhang Y. et al., 2020). Based on the barriers of exosomes as drug carriers, Yanzhen Yu and colleagues developed a functional ECM biomaterial capable of loading exosomes, which is able to capture and enrich exosomes without damaging the exosome membrane, increasing the stability of exosomes as carriers for delivery of anti-PF miR-29. The investigators observed that the application of this miR-29 exosome-enriched ECM material in bleomycin-induced PF mice resulted in good attenuation of lung tissue fibrosis in mice, which provided inspiration for optimizing the exosome delivery technology (Yu et al., 2021). Therefore, as the technology continues to improve, exosomes may hold a promising future as anti-PF agent delivery vehicles.

## 6 Conclusion

In recent years, research on exosomes has increased rapidly. In this context, many advances have been made in the study of the relationship between exosomes and a variety of respiratory diseases, including PF. Exosomes play an important role in communication between human cells, can participate in multiple physiopathological processes, and are closely associated with many therapeutic mechanisms (Guiot et al., 2019; Mashouri et al., 2019; Soraya et al., 2021; Yamada, 2021). According to several studies mentioned above, exosomes have the ability to participate in processes such as inflammation and immunity, ECM deposition, and EMT in PF, which is associated with the ability of exosomes to regulate inflammatory cell infiltration, immune cell proliferation, and fibroblast proliferation and differentiation in the lung, as well as to influence cytokines such as TGF-β1. Exosomes have great potential for the diagnosis and determination of prognosis in PF and show therapeutic promise for the prevention, mitigation, and even reversal of PF. In addition, the biocompatibility, low immunogenicity, and ability to target tissues of exosomes provide new insights into the development of anti-PF drug delivery systems.

However, there is still a long way to go to translate the potential of exosomes as biomarkers and therapeutics in PF into clinical applications that can benefit patients. The diagnostic and therapeutic mechanisms of exosomes in PF are currently unclear and need to be further investigated. The technical basis for the clinical application of exosomes is immature due to obstacles such as exosome extraction, preservation, and drug loading. As highlighted at the 2021 annual meeting of the International Society for Extracellular Vesicles, clinical applications of EVs such as exosomes need to explore effective, rapid, simplified, reproducible, and scalable pathways. Clinical translation of current results and further achievements require continuous efforts of multidisciplinary teams (Picciolini et al., 2021). Despite these obstacles, the prospect of clinical application of exosomes in PF currently requires more efforts to be invested in the research of exosomes as a diagnostic method and new therapies for PF to benefit PF patients.
